# Expert opinion on management of pancreatic exocrine insufficiency in pancreatic cancer

**DOI:** 10.1016/j.esmoop.2022.100386

**Published:** 2022-02-03

**Authors:** G. Roeyen, F. Berrevoet, I. Borbath, K. Geboes, M. Peeters, B. Topal, E. Van Cutsem, J.-L. Van Laethem

**Affiliations:** 1 Department of Hepatobiliary Transplantation and Endocrine Surgery, Antwerp University Hospital and University of Antwerp, Edegem; 2 Department of General and Hepatobiliary Surgery, Ghent University Hospital, Ghent; 3 Hepato-Gastroenterology Unit, Cliniques Universitaires Saint-Luc, Brussels; 4 Department of Gastroenterology, Division of Digestive Oncology, Ghent University Hospital, Ghent; 5 Department of Oncology, Antwerp University Hospital and University of Antwerp, Edegem; 6 Department of Visceral Surgery, University Hospitals KU Leuven, Leuven; 7 Department of Gastroenterology/Digestive Oncology, University Hospital Leuven, University Hospitals Gasthuisberg Leuven and KU Leuven, Leuven; 8 Department of Digestive Oncology, University Hospital Erasmus Brussels, Erasme University Hospital, Université Libre de Bruxelles, Brussels, Belgium

**Keywords:** pancreatic cancer, pancreatic exocrine insufficiency, pancreatic enzyme replacement therapy

## Abstract

Pancreatic exocrine insufficiency (PEI) is a common condition in patients with pancreatic cancer (PC). PEI can be due to the tumor, which, if located in the head, causes obstruction of the pancreatic duct with subsequent atrophy of the pancreatic parenchyma, or it can be the consequence of pancreatic surgical resection. The standard treatment of PEI is pancreatic enzyme replacement therapy (PERT). Clinical data to support the use of PERT in PC are however limited. There are very few randomized clinical trials that evaluated PERT in PC. Most data come from observational studies. Despite this limited clinical evidence, PERT treatment for PEI is an essential part of supportive therapy to ensure optimal nutritional status in PC patients who will receive surgery, neoadjuvant/adjuvant or palliative treatment. The objective of this review is to increase the awareness about PEI in PC patients and to provide expert recommendations on the use of PERT in resected, borderline resectable and unresectable patients, based on clinical experience and literature review.

## Highlights

• PEI is a common condition in PC patients and should be actively looked for in PC patients and treated with PERT.
• In resected and borderline resectable patients PERT should be started as soon as possible when PEI is clinically suspected.
• Early PERT start is key to have resected and borderline resectable patients as fit as possible before cancer treatments.
• In unresectable patients, a confirmation of PEI via an indirect test is recommended before PERT initiation.
• A cornerstone of PERT is proper patient education on its use and adequate patient follow-up to adapt to therapy as needed.

## Introduction

Within Europe, pancreatic cancer (PC) has been found to be the fourth most fatal cancer in men, after lung, colorectal and prostate cancer and in women after breast, colorectal and lung cancer.^1^^,^^2^ Among major cancer locations, PC remains the only one showing no overall falls in mortality rate over the last two decades in the European Union.^3^ In Belgium, a rise in incidence rate (*n*/100 000 person-years) was noted between 2005 and 2019 from 5.9 to 9.1 in men and from 4.6 to 7.2 in women, with 1041 new cases in men and 987 in women in 2019.^4^ The majority of patients with PC will present with either metastatic or locally advanced cancer. Surgical excision followed by adjuvant chemotherapy is the only definite treatment with a 5-year survival of ∼20%. Resection however is only possible in 15%-20% of the PC patients. Defining the treatment strategy for patients suffering from PC requires a specialized multidisciplinary team that includes hepato-pancreato-biliary surgeons, medical oncologists, gastroenterologists, radiation oncologists, radiologists, pathologists, dietitians and supportive and palliative care specialists.^2^ An important aspect of best supportive care is nutritional support. At the time PC patients present themselves, their nutritional status is often poor; 30% of the patients are malnourished, 80% report weight loss and over a third has lost >10% of their initial body weight.^5^ Malnutrition and weight loss in cancer patients can be explained by different mechanisms such as changes in tumor cell metabolism, loss of appetite and tumor-derived factors, which all contribute to cachexia.^6^ In PC patients, pancreatic exocrine insufficiency (PEI) is an additional important contributor to malnutrition.^7^

To guide clinicians where formal guidelines are not available, we provide herein, based on our clinical experience together with data from the literature, recommendations on the use of PERT in different settings of PC.

## Pancreatic exocrine insufficiency

Multiple definitions of PEI have been used throughout the literature. One definition often cited is ‘an insufficient secretion of pancreatic enzymes and bicarbonate to maintain a normal digestion’.^8^ Another definition is ‘a condition in which the amount of secreted pancreatic enzymes is not enough to maintain normal digestion’.^9^

PEI in PC can be tumor induced, through obstruction of the main pancreatic duct, fibrosis of the gland and loss of pancreatic exocrine tissue, or can be the result of surgical removal of pancreatic tissue.^10^^,^^11^ Patients may thus experience PEI before diagnosis, during non-surgical treatment and following surgery.^6^

The most common clinical symptoms of PEI are diarrhea, steatorrhea, voluminous and foul smelling stool, abnormal stool frequency, bloating, abdominal discomfort/pain, excess flatulence and weight loss.^12^ Symptoms alone, however, may not be sufficient to diagnose PEI as they vary amongst patients and the individual symptoms can be due to other comorbidities or cancer treatment. Steatorrhea can also be caused by diseases affecting the small intestine, such as bacterial overgrowth. Diarrhea after resection also can have another etiology than PEI; for example, removal of periarterial neural tissue can result in altered motoric bowel function. Diarrhea and maldigestion can also be the results of chemotherapeutic treatment. A differential diagnosis, specifically in PC context, is therefore needed to exclude conditions other than PEI that could lead to similar symptoms (see Table 1).

**Table 1:** Symptoms common between PEI and other conditions in pancreatic cancer patients

Symptom	PEI^12^	Surgically altered anatomy/denervation	SIBO	Chemotherapy	Cancer cell metabolic factors
Diarrhea	++	++	++	++	
Steatorrhea	++		++		
Voluminous and foul smelling stool	++		++		
Abnormal stool frequency	+	++	+	+	
Excess flatulence	+		+	+	
Abdominal discomfort/pain	+	+	+	+	
Abdominal bloating	+		+	++	
Malnutrition/weight loss	++		+	+ (due to nausea)	++
Fat-soluble vitamin deficiencies	+				

+, often, not always; ++, very frequent; PEI, pancreatic exocrine insufficiency; SIBO, small intestinal bacterial overgrowth.

In the case that confounding factors exist, it is advisable to diagnose PEI not based on symptom evaluation only, but in conjunction with a reliable test.^13^ A proper diagnosis is important, since untreated PEI will lead to maldigestion, malabsorption and malnutrition, and as a consequence patients will develop also fat-soluble vitamin deficiencies resulting in a variety of secondary symptoms.^7^^,^^13^

## Testing for PEI

Both direct and indirect tests to diagnose PEI can be used.^6^^,^^13^^,^^14^ Direct tests that measure the release of pancreatic juice in the duodenum have a good specificity and sensitivity but are invasive, time-consuming and expensive. Therefore, in clinical practice, indirect tests are used (see Table 2). Steatorrhea, an important clinical symptom of PEI, can be assessed by measuring the fecal fat in stool and calculation of the coefficient of fat absorption (CFA). The fecal fat test is considered the gold standard to detect malabsorption.^15^ It is however experienced by patients as an unpleasant test. Stools have to be collected during 3 days and patients have to be on a strict diet containing 100 g of fat per day over a 5-day period, which causes abdominal discomfort.^16^

**Table 2:** Advantages and limitations of indirect tests for PEI diagnosis

	CFA	FE-1	Breath test	Nutritional markers
Advantages	Gold standard to measure malabsorption of fat	Test widely available	Directly measures fat digestion	Blood testing widely available
		Specificity: 88%; Sensitivity: 92.9%	Specificity: 91.7%; Sensitivity: 92.9%	
		Simple: can be measured in a single stool sample	Accurate to diagnose PEI after surgery	
Limitations	Experienced as unpleasant	Less accurate to diagnose mild PEI	Test not widely available	Needs further research and validation
	High-fat diet required for 5 days, with 3 days stool collection	Less accurate to diagnose PEI after surgery	Long test period (6 h)	
	No distinction between liver, intestinal or pancreatic causes of malabsorption	Proteolytic enzyme (not lipolytic)		
		Unreliable in case of loose stools		

CFA, coefficient of fat absorption; FE-1, fecal elastase-1; PEI, pancreatic exocrine insufficiency.

In addition, the pancreas has a large functional reserve and clinically evident PEI occurs only when 90% of the function is lost and the secretion of pancreatic enzymes is <10% of normal.^17^ Therefore, this test may not be useful to detect earlier stages of PEI. Moreover, the CFA is a non-specific index for assessing fat malabsorption and cannot differentiate between liver, intestinal or pancreatic causes.^13^

To evaluate PEI, tests other than the CFA test may be more useful. The indirect test that is mostly used and widely available is the fecal elastase-1 (FE-1) test, which measures FE-1.^18^^,^^19^ Elastase-1 is a proteolytic enzyme produced by pancreatic acinar cells, which passes through the gut with only a minor degree of degradation. It therefore can be quantified in fecal samples and is a measure for exocrine enzyme activity in general. It is not a measure for lipolytic activity. There is no consensus on the cut-off point for PEI diagnosis, yet an arbitrary cut-off of <200 μg/g is often used. A systemic review and meta-analysis found a sensitivity of 77% and a specificity of 88%.^20^ The sensitivity to diagnose mild PEI was found to be only 47% in this meta-analysis. Other research groups also reported the FE-1 test to be less accurate after pancreatic resection.^21^, ^22^, ^23^ Another limitation is that the test is unreliable when stools are loose.^16^

Another indirect test to measure PEI is the ^13^C-mixed triglyceride breath test consisting of an oral administration of a ^13^C marked test meal. The fat present in the test meal will get hydrolyzed in proportion to the amount of pancreatic lipase activity. The amount of ^13^CO_2_ measured in breath samples during a 6-hour testing period after intake of the test meal reflects the absorption and metabolization of the triglycerides. A protocol often referred to in the literature reports a cumulative recovery rate of <29% used as a cut-off for diagnosis.^24^ A sensitivity of 92.9% and a specificity of 91.7% have been reported for this test. Following pancreatic surgery, the breath test was reported to be more accurate than the FE-1 test.^23^ A recent review considers the breath test as likely the indirect test nearest to a gold standard currently available.^13^ At present, this test is not widely available. In Belgium, the breath test is available since many years and is well established and widely used to diagnose PEI, using a cumulative recovery rate of <23% for diagnosis.^25^ Because of its higher specificity and sensitivity, and if available in the country or medical center, we recommend the breath test as an indirect method to diagnose PEI.

Finally, assessment of serum nutritional markers can also be used to predict the probability of PEI and provide guidance on PERT use. More specifically, a decrease in magnesium, albumin, pre-albumin and retinol-binding protein levels has been proposed as a predictive model for the diagnosis of PEI and for monitoring the response to treatment.^26^ Also deficiencies in the fat-soluble vitamins A, D and E may be indicative of PEI.^27^

## Prevalence of PEI in PC

Figures on the prevalence of PEI reported in the literature show that it is a common condition in both resectable and unresectable PC. There is however a wide variety in the numbers reported, probably due to different definitions, research settings and testing methods. Whereas in case of total pancreatectomy the prevalence of PEI will be 100%, the prevalence after other types of surgery varies. A systematic review evaluating PEI in patients with PC before and after pancreaticoduodenectomy (PD) found the prevalence of PEI pre-operatively to be 44% and post-operatively 74% (range 36%-100%).^28^ In case of distal pancreatectomy (DP), PEI was present in 20% of patients pre-surgery and in 67%-80% after DP. According to another review, PEI after PD ranges from 70% to 100%.^29^ A lower prevalence was found in patients who had undergone DP (30%-66%). This review also reported a substantial prevalence pre-operatively ranging from 46% to 100%.

A Belgian prospective cohort study in patients who had to undergo PD for an oncological indication reported a PEI prevalence of 20% pre-operatively and 64.1% post-operatively.^30^ Results from a not-yet published sub-analysis that took only pancreatic adenocarcinoma patients into account (*n* = 159) showed a PEI prevalence of 26.6% before PD and 78.1% after PD. In the case of DP (*n* = 39), the prevalence of PEI was 15% pre-operatively and 10% post-operatively.

PEI is also common in unresectable cancer with a prevalence ranging from 50% to 100%.^29^ A recent meta-analysis showed that PEI was prevalent in 72% of unresectable PC, and that PEI was 3.4 times more prevalent in patients with pancreatic head versus pancreatic tail tumors.^31^ Of note, 78% of PC tumors are located in the head, which contains the largest part of the exocrine tissue. The remaining 22% of PC tumors are equally distributed in the body and in the tail.^32^ PEI also seems to be evolving during the disease. A prospective study in unresectable cancer showed incidences of PEI between 66% and 92% and a decline of the pancreatic exocrine function of ∼10% per month.^33^

## Pancreatic enzyme replacement therapy

Together with dietary advice, pancreatic enzyme replacement therapy (PERT) is the standard treatment for PEI.^34^ The objective of the therapy is to deliver sufficient enzymatic activity into the duodenal lumen together with the meal in order to restore nutrient digestion and absorption. The aim is therefore to mimic pancreatic physiology as much as possible. To prevent the inactivation of lipase by gastric acid, PERT is encapsulated in microgranules or minimicrospheres with a pH-sensitive coating. The size of the particles is <2 mm, to ensure fast emptying in the duodenum.^35^ PERT consists of a mixture of pancreatic enzymes and needs to be taken together with each meal and snack. There are several guidelines with different recommendations regarding PERT dosing. All recommend a dose well beyond the physiological lipase output, which in healthy individuals is estimated to be between 3 60 000 and 9 60 000 units after a standard meal.^11^ As steatorrhea only occurs when the lipase output falls to <10% of normal, the minimum number of lipase units required for normal digestion is thought to be lower. The European working group on chronic pancreatitis recommends a minimum lipase dose of 40 000-50 000 units with main meals, and half the dose with snacks.^35^ The International Study Group on Pancreatic Surgery recommends to start therapy after pancreatic resection with doses of 40 000-50 000 units of lipase per meal and 10 000-25 000 units with every snack.^9^ They precise that dose escalation and inhibition of gastric acid secretion may be warranted according to response. Some guidelines recommend higher starting doses. A Spanish surgery guideline mentions higher starting doses of 72 000-75 000 units with main meals and 36 000-50 000 units with snacks.^15^ Also, for unresectable PC, a Spanish consensus statement recommends a higher dose of minimal 75 000 units per meal.^36^

Several formulations of PERT have been developed and made commercially available. In Belgium, only one medicinal product is available, based on a porcine pancreatic enzyme extract (containing lipase, amylase and protease), encapsulated in enteric-coated mini-microspheres sized between 0.7 and 1.6 mm.^37^ It is available in a formulation of 10 000, 25 000 or 35 000 units of lipase per capsule.

The overall safety profile of pancreatic enzymes is similar across all formulations.^38^ The most common adverse effects are gastrointestinal in nature (e.g. abdominal pain, abdominal distension, diarrhea, constipation, vomiting, nausea) and generally mild to moderate in severity; some may be symptoms of the underlying disorder rather than a treatment effect. Allergy and hypersensitivity reactions, mainly skin rash, have been reported, but occur rarely.

## Guidance on PERT use in PC

Although treatment of PEI with PERT seems logical, the clinical evidence to support PERT use in PC is limited. Most data regarding the efficacy of PERT originate from the pancreatitis research field.^39^ As will be discussed further in this review, very few randomized controlled trials (RCTs) have been carried out in the setting of PC and results are sometimes conflicting. Recommendations and decisions on the use of PERT therefore have to be taken in the absence of robust clinical evidence. Some countries (e.g. Australia, UK, The Netherlands) have already issued specific guidelines on the use of PERT in PC.^40^, ^41^, ^42^ Other countries, like the UK, have taken a pragmatic approach and recommend to give PERT to all PC patients without the need for diagnostic proof of PEI. Also, the International Study Group on Pancreatic Surgery recommends to initiate PERT routinely for a period of 6 months after DP.^9^ In Belgium, there is at present no specific guidance on PERT use in PC. However, since very recent times, PERT is completely reimbursed for all PC patients with PEI.

## Recommendations for PERT in resected PC patients

Based on our clinical experience and the data on prevalence found in the literature, the likelihood for PEI following pancreatic surgery is very high, especially after PD. Patients should be carefully monitored for clinical symptoms that are indicative of PEI. The probability of the patient to develop PEI also can be judged based on the location of the tumor, the type of surgery carried out or presence of atrophy in the post-operative pancreatic remnant as shown in pre-operative imaging reports. Also, assessment of the nutritional status may help to identify patients who are likely to have PEI. In case of doubt, PEI needs to be diagnosed with an appropriate test. As described earlier, PEI can be present already pre-operatively, especially when the pancreatic head is involved. In case of suspicion, it is recommended to test for PEI already before surgery.

PERT is the standard therapy for PEI, and it seems logical to treat PEI in resected PC patients with PERT. However, clinical evidence on the effectiveness of PERT after resection is limited: only two RCTs could be identified. In one RCT, where the indication for surgery was chronic pancreatitis or PC, the efficacy of PERT in improving malabsorption symptoms (improvement of fat and protein digestion and increase in body weight) after pancreatic surgery was shown.^43^ More recently, an RCT was conducted in Korea to study the effect of PERT on body weight nutritional status and quality of life (QoL) after PD.^44^ In the per-protocol analysis, but not in the intention-to-treat analysis, a significant body weight gain was observed in the PERT group. Since non-compliance with medication was the main reason for not having completed the study as per protocol, the authors concluded that their finding emphasizes the importance of medication compliance. No difference in QoL was observed between the PERT and placebo group.

One observational study in resectable periampullary cancer patients undergoing PD did suggest that PERT use was associated with improved survival (33.1 versus 26.7 months, *P* = 0.034).^45^

As clinical data are limited, a clear need for large well-designed RCTs remains.

We recommend that PERT is initiated as soon as PEI is diagnosed based on likelihood and/or clinical symptoms. Only in case of doubt because of confounding co-existing conditions, PEI should be confirmed via an indirect test.

The aim of PERT in this group of resected patients is to relieve symptoms of malabsorption and improve nutritional status, and to have the patients in a condition as fit as possible before the start of adjuvant chemotherapy. The best practice should therefore be that PERT is initiated by the pancreatic surgeon and that the patient is on treatment by the time the oncologist initiates chemotherapy.

## Recommendations for PERT in patients with borderline resectable PC

No specific data on PEI prevalence in borderline resectable PC were identified.

However, as data from the literature show that PEI can be already present before resection, it is logical to assume that PEI can also be prevalent in borderline resectable patients, especially when the pancreatic head is involved. In order to have the patients as fit as possible to undergo neoadjuvant therapy, we recommend that an empirical trial of pancreatic enzyme replacement is started based on patient history and/or clinical symptoms. In case of doubt due to confounding co-existing conditions, PEI can be at a later stage confirmed via an indirect test.

## Recommendations for PERT in patients with unresectable PC

The current practice in Belgium is not to test routinely for PEI in unresectable PC patients, which could be either locally advanced or metastatic patients. Whether PEI will develop will depend on the characteristics of the primary tumor and already existing atrophy of the pancreas parenchyma. In metastatic PC patients, for example, the primary tumor is sometimes very small or located in the pancreatic tail. If a sufficient amount of pancreatic tissue is preserved, it is unlikely that these patients will develop PEI.

Concerning the clinical benefit, only a few RCTs have been carried out in unresectable PC patients, with conflicting results. It should be noted that these trials were very small, designs were very heterogeneous, primary endpoints were different and the randomization protocol was applied regardless of PEI status. In one RCT, PERT was found to improve nutritional status and increase body weight.^46^ Another RCT could demonstrate a greater weight loss in the placebo group, but did not find a statistical difference in body mass index (BMI), nutrition score or QoL.^47^ One RCT could not find a benefit when looking at reduction of weight loss, QoL or survival.^48^ Another RCT did not show a difference between the PERT and placebo group in change of BMI or levels of serum nutritional markers.^49^

Data coming from observational studies also suggest a benefit for PERT in unresectable PC for survival. A retrospective analysis conducted in Spain suggested that PERT conferred a survival advantage to those with unresectable PC receiving PERT (189 versus 95 days, *P* < 0.001).^34^ The authors noted that the proportion of patients in the study who were suitable for chemotherapy and the number of therapy cycles that these patients tolerated increased with PERT.

A recent population study in the UK also reported that PERT was independently associated with longer survival in those with unresectable PC regardless of chemotherapy [survival time ratio: 3.46, 95% confidence interval (CI) 1.99-5.99].^50^

Finally, a meta-analysis that included, besides the previously discussed four RCTs, also eight prospective and retrospective observational studies could show a benefit in terms of survival. PERT was associated with 3.8 (95% CI 1.37-6.19; *P* = 0.002) months’ survival benefit and improvement of body weight.^31^ A meta-analysis that included only the four RCTs however could not find a significant difference for overall survival, change in weight or QoL.^51^

As for resected PC, there is a need for large well-designed randomized clinical studies to understand the benefit of PERT in unresectable PC. Encouraging results came from a recent prospective non-randomized pilot study that was conducted as a feasibility study to instruct further larger clinical trials. The study looked at effects of PERT on symptoms using the general quality of life questionnaire- C30 (QLQ-C30) questionnaire and the PC-specific quality of life questionnaire-Pan26 (QLQ-Pan26) questionnaire. The authors found that PERT was able to reduce diarrhea and bloating/gas symptoms.^52^ The setup of this study may help the design of larger RCTs in the future.

Although strong clinical evidence for the benefit of PERT is lacking, we believe that clinical signs of PEI should be actively looked for in unresectable patients. Since there may be a variety of reasons for clinical symptoms like weight loss and diarrhea in patients with advanced PC, installing PERT only based on clinical symptoms seems less appropriate. If possible, and if not jeopardizing start of potential systemic treatments, it is advised to confirm the diagnosis of PEI by carrying out an indirect test.

In case PEI is diagnosed, PERT needs to be started. Interventions to provide relief from malnutrition are part of palliative and supportive care.^2^ Patients with unresectable PC have a very bad prognosis with a 5-year survival of only 13% for regional and 3% for metastatic PC.^53^ In patients with PC, the development of PEI, as measured by the level of FE-1, was shown to be an independent predictor of survival in advanced PC.^54^

The goal of PERT in this patient group is to increase QoL and possibly survival. A qualitative inquiry framework study revealed that a major QoL concern for patients and their carers/family was difficulty in managing gut symptoms and complex dietary issues.^55^ Issues were related to lack of information about malabsorption and managing symptoms of PEI.

According to a recent review on PC-associated weight loss by the Precision Promise Consortium from the Pancreatic Cancer Action Network in the United States, slowing down weight loss in patients with PC may improve quality and length of life both directly, through maintaining function, and indirectly, through enhancing therapeutic tolerance.^56^ When PEI is present, PERT may help to decrease weight loss. They recommend therefore that patients with evidence or suspicion of PEI should be initiated on pancreatic enzymes.

Finally, we also would like to emphasize that the optimization of the nutritional and performance status is considered important in order to make patients with unresectable PC as fit as possible and eligible for new treatment options. It can be assumed that PERT also will play a role here.

## Recommendations for PERT dosing

We consider that 40 000-50 000 units of lipase to be taken with each meal and 25 000 units to be taken with each snack is appropriate as initial dosing. This is in line with the European guidelines for chronic pancreatitis and a recent review paper on PERT in PC.^6^^,^^35^

Patients can have different degrees of PEI and the dose may need to be adapted to the need of the individual patients. For example, our experience is that patients who underwent a complete pancreatectomy need higher starting doses of PERT.

No specific marker is used to assess the efficacy of the adequate dosing. Adequacy of the initial dose is to be judged on the basis of rapid improvement of clinical symptoms. The efficacy of PERT can be evaluated based on the relief of maldigestion-related symptoms (e.g. steatorrhea, weight loss, flatulence) and the normalization of the nutritional status of the patients. Evaluation of the nutritional status of patients can be based on anthropometric and analytical parameters, including biochemical results for oligo-elements, liposoluble vitamins, and lipoproteins. Some authors have proposed that measurements of magnesium, albumin, pre-albumin and retinol-binding protein levels can be used as a panel to monitor the response to PERT.^26^

**Figure 1:**
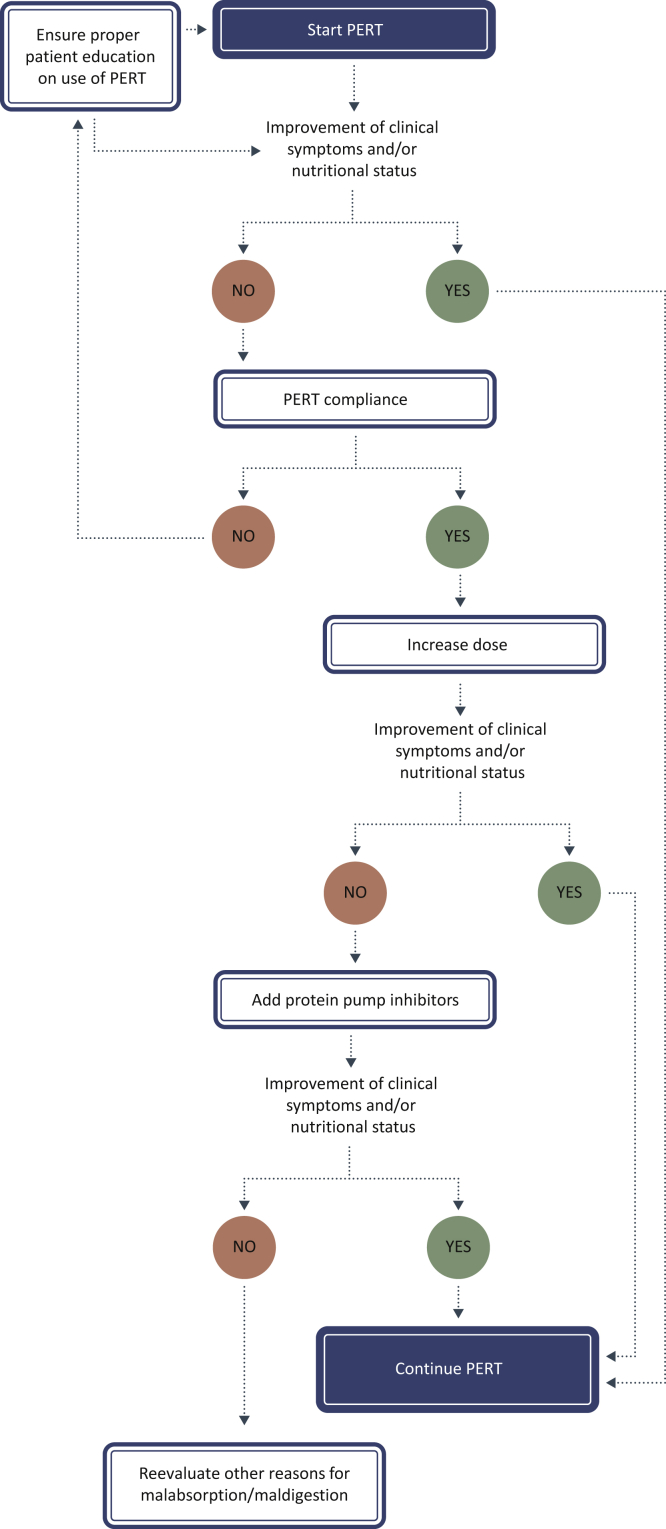
**Recommendations for patient follow-up on PERT use.** PERT, pancreatic enzyme replacement therapy.

It is important to collaborate with dietitians to ensure correct and optimal use of PERT by the patients and to provide specific dietary counseling. In Figure 1, a recommendation for patient follow-up is provided. In case no improvement in clinical symptoms or nutritional status is observed, the first step should be to check PERT compliance and appropriate use. If this is under control and symptoms persist, the dose of PERT should be increased rapidly in order to establish the ideal dosing. Also, the use of proton pump inhibitors to avoid PERT inactivation by gastric acids should be considered to improve the response to PERT.^57^

## Optimizing PEI diagnosis and treatment

Data from several countries have indicated that there is under-diagnosis and/or under-treatment in patients with PC.^58^^,^^59^ Recent data from a large population study in the United States showed that only 1.9% of patients with PC had testing for PEI, 21.9% of patients were treated and 90% received sub-therapeutic doses.^60^ In the UK, despite the National Institute for Health and Care Excellence recommendation to give PERT to all PC patients, only 74.4% of patients with potentially resectable disease and 45.3% of patients with unresectable disease were prescribed PERT.^61^ No specific data exist for Belgium on the use of PERT in PC. We estimate however that it is likely that also there is both under-diagnosis and under-treatment of PEI. Efforts should be made to raise awareness about PEI and the importance to diagnose PEI and treat with PERT. We recommend to apply a low threshold for considering PEI in all PC patients presenting with symptoms of malabsorption. Still, if doubts exist about the diagnosis, a functional test to confirm PEI should be carried out. If PEI is diagnosed, prescription of PERT should be ensured and patients should get educated properly on how to use PERT. A regular patient follow-up needs to be foreseen to ensure adequate usage and dosing.

## Conclusion

**Figure 2:**
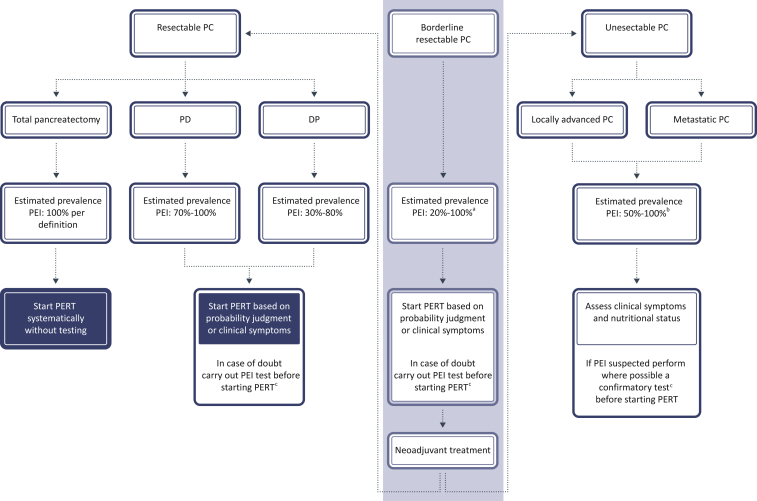
**Practical approach for PEI diagnosis and PERT use in PC.** DP, distal pancreatectomy; PC, pancreatic cancer; PD, pancreaticoduodenectomy; PEI, pancreatic exocrine insufficiency; PERT, pancreatic enzyme replacement therapy. ^a^Based on literature data on pre-surgery prevalence in resectable PC. ^b^Combined (locally advanced and metastatic) data in the literature. ^c^In Belgium, this is the ^13^C triglyceride breath test.

PEI is a condition that is highly prevalent in both resected and unresectable PC patients. Recommendations on diagnosis of PEI and the use of PERT in different settings of PC are depicted in Figure 2. In our opinion, most of the patients who undergo PD will need PERT. After resection, PERT should be installed based on the likelihood of PEI and/or the presence of clinical symptoms. Also in borderline resectable PC patients, PERT should be initiated based on clinical symptoms in order to have them as fit as possible to receive neoadjuvant treatment and surgery. A differential diagnosis is however important in both settings to exclude other conditions that could give rise to symptoms similar to those associated with PEI. In case of doubt, an indirect test should be carried out to confirm the diagnosis of PEI. In unresectable patients with advanced PC, we recommend to test systematically for PEI via an indirect test, since multiple factors may be at the origin of maldigestion and malnutrition in these patients. Pending on the availability in the country, preference should be given to the ^13^C-mixed triglyceride breath test as an indirect test method.

The currently available clinical data mainly involve observational studies and are not sufficient to support a more general use of PERT in all PC patients. We also stress the importance of a proper education and strict follow-up of patients to ensure the adequate use and dosing of PERT.

In order to make more evidence-based decisions on the use of PERT in the different settings of PC in the future, it is important that additional clinical research be carried out.
